# Supplementary material for: Role and mechanism of NCAPD3 in promoting malignant behaviors in gastric cancer
Source: Front Pharmacol. 2024 Apr 22;15:1341039. doi: 10.3389/fphar.2024.1341039 (PMC11070777; doi:10.3389/fphar.2024.1341039)
Supplement: Supplementary file 11 [file DataSheet2.ZIP › GSEA/Oncogenic signature/my_analysis.Gsea.1599461962066/ESC_J1_UP_LATE.V1_UP.html]

Details for gene set ESC\_J1\_UP\_LATE.V1\_UP[GSEA]

|  || Dataset | filtered\_dataset.sample\_info.cls#WT\_versus\_NCAPD3\_MUT |
| Phenotype | sample\_info.cls#WT\_versus\_NCAPD3\_MUT |
| Upregulated in class | NCAPD3\_MUT |
| GeneSet | ESC\_J1\_UP\_LATE.V1\_UP |
| Enrichment Score (ES) | -0.42337558 |
| Normalized Enrichment Score (NES) | -1.92945 |
| Nominal p-value | 0.007352941 |
| FDR q-value | 0.030885 |
| FWER p-Value | 0.211 |
Table: GSEA Results Summary

  

Fig 1: Enrichment plot: ESC\_J1\_UP\_LATE.V1\_UP      
 Profile of the Running ES Score & Positions of GeneSet Members on the Rank Ordered List

  

| SYMBOL | TITLE | RANK IN GENE LIST | RANK METRIC SCORE | RUNNING ES | CORE ENRICHMENT || 1 | 57190 | SEPN1 | 300 | 0.591 | -0.1603 | No |
| 2 | 2244 | FGB | 321 | 0.582 | -0.1202 | No |
| 3 | 1601 | DAB2 | 327 | 0.577 | -0.0698 | No |
| 4 | 57619 | SHROOM3 | 783 | 0.331 | -0.3659 | No |
| 5 | 7429 | VIL1 | 827 | 0.299 | -0.3687 | No |
| 6 | 4616 | GADD45B | 904 | -0.330 | -0.3924 | Yes |
| 7 | 4008 | LMO7 | 909 | -0.335 | -0.3639 | Yes |
| 8 | 4645 | MYO5B | 985 | -0.395 | -0.3809 | Yes |
| 9 | 7286 | TUFT1 | 1010 | -0.412 | -0.3595 | Yes |
| 10 | 1604 | CD55 | 1090 | -0.468 | -0.3725 | Yes |
| 11 | 5097 | PCDH1 | 1132 | -0.502 | -0.3550 | Yes |
| 12 | 1282 | COL4A1 | 1165 | -0.534 | -0.3280 | Yes |
| 13 | 8535 | CBX4 | 1184 | -0.555 | -0.2890 | Yes |
| 14 | 2152 | F3 | 1188 | -0.559 | -0.2388 | Yes |
| 15 | 58480 | RHOU | 1189 | -0.560 | -0.1864 | Yes |
| 16 | 6690 | SPINK1 | 1200 | -0.577 | -0.1395 | Yes |
| 17 | 22822 | PHLDA1 | 1255 | -0.643 | -0.1181 | Yes |
| 18 | 80201 | HKDC1 | 1289 | -0.681 | -0.0781 | Yes |
| 19 | 54463 | FAM134B | 1356 | -0.815 | -0.0492 | Yes |
| 20 | 5156 | PDGFRA | 1383 | -0.932 | 0.0194 | Yes |
Table: GSEA details [plain text format]

  

Fig 2: ESC\_J1\_UP\_LATE.V1\_UP      
 Blue-Pink O' Gram in the Space of the Analyzed GeneSet

  

Fig 3: ESC\_J1\_UP\_LATE.V1\_UP: Random ES distribution      
 Gene set null distribution of ES for **ESC\_J1\_UP\_LATE.V1\_UP**
